# Impact of the Spanish smoke-free laws on cigarette sales by brands, 2000–2021: Evidence from a club convergence approach

**DOI:** 10.18332/tid/174407

**Published:** 2023-12-04

**Authors:** Miguel Ángel Del Arco-Osuna, Josep Blasco, Alejandro Almeida, Juan Manuel Martín-Álvarez

**Affiliations:** 1 Department of Quantitative Analysis for Economics and Management, Universidad Internacional de La Rioja, Logroño, Spain

**Keywords:** cigarette sales, club convergence, smoke-free laws, Spain, time series

## Abstract

**INTRODUCTION:**

In January 2006, the Spanish government enacted a tobacco control law that banned the advertising, promotion and sponsorship of tobacco. In January 2011, further legislation on this matter was adopted to provide a more restrictive specification of the ban. In this study, we analyze the effect produced on cigarette sales by these two prohibitions. We address this problem using a cluster time-series analysis to test whether the sales of cigarettes by brands have been homogenized with the prohibition of advertising, promotion, and sponsorship.

**METHODS:**

The data source used was the official data on legal sales of cigarettes by brands in Spain, from January 2005 to December 2021 (excluding the Canary Islands and the Autonomous Communities of the cities of Ceuta and Melilla). To achieve our objective, we used log(t) test statistics to check if there is global convergence in the three selected periods according to the regulatory changes that have occurred in Spain (2005–2021, 2005–2010 and 2011–2021). Second, once absolute convergence is rejected, we applied a clustering algorithm to test for the existence of subgroup convergence.

**RESULTS:**

The cigarette brands that have been marketed during the period 2005–2021 (n=40), can only be grouped into three groups according to the behavior of their sales. When we focus on the period 2005–2010 (n=74), cigarette brands are grouped into five groups according to their sales behavior. Finally, the cigarette brands marketed during the period 2011–2021 (n=67) are grouped into three groups according to the temporal evolution of their sales. These results suggest a greater homogenization of cigarette sales after the application of the law of January 2011.

**CONCLUSIONS:**

Act 42/2010 (total ban on tobacco advertising, promotion, and sponsorship actions) was associated with greater homogenization of cigarette sales than the application of Act 28/2005 (partial ban). This finding supports what is established in the previous literature that indicates that Act 42/2010 provided a more restrictive specification of the ban than Act 28/2005.

## INTRODUCTION

It is well known and widely studied that smoking causes acute and chronic diseases^1^. To minimize this harmful effect on the health of the population, the Framework Convention of the World Health Organization for Tobacco Control (WHO-FCTC)^2^ is the first international health treaty that requires that countries implement tobacco control measures. In Spain, the WHO-FCTC was ratified on 11 April 2005. In 2008, the WHO developed the MPOWER package^3^ to help nations meet their commitments to the treaty. One of the six components of MPOWER is to enforce bans on tobacco advertising, promotion, and sponsorship.

To minimize the impact that tobacco use has on society, governments have taken measures that affect both demand and supply. On the demand side, laws prohibiting smoking or considering the impact of taxes on prices have been passed^4^. On the supply side, tobacco product manufacturers and retailers have been limited to maintaining prices to promote tobacco products and keep demand high^5,6^. These measures arise because the WHO-FCTC has accelerated the implementation of measures in several crucial political domains, one of which is the ban on tobacco advertising, promotion, and sponsorship^7^.

In the case of Spain, in recent years the government has successfully applied different restrictive laws to control the legal sale of cigarettes. Specifically, the Spanish anti-tobacco laws of 2005 and 2010 affected consumption by introducing restrictions on the demand and supply side, with Act 28/2005 (December 26)^8^ and Act 42/2010 (December 30)^9^, with health measures against smoking and regulation of the sale, supply, consumption, and advertising of tobacco products. Both laws introduce a harsh restriction on marketing strategies, since the advertising, promotion and sponsorship of tobacco is not allowed outside of an establishment authorized for the retail sale of tobacco under a monopoly regime. Specifically, the text of Act 28/2005 indicates that ‘the sponsorship of tobacco products is prohibited, as well as all kinds of advertising, and promotion of the aforementioned products in all media and supports, including vending machines and information society services’. These measures seek to homogenize the market and make tobacco less attractive, especially to the young population. Although the advertising, promotion and sponsorship of tobacco were banned under Act 28/2005, Act 42/2010 provided a more restrictive specification of the ban^10,11^.

The effect that the laws have had on the Spanish tobacco market since 2005 have been analyzed from different perspectives. On the one hand, there are studies that expose the effects on the prevalence of smokers and tobacco sales^6,11,12^. On the other hand, other studies have focused on exposure to environmental tobacco smoke^13,14^. However, few of them focus on analyzing compliance with the bans on tobacco advertising, promotion, and sponsorship^7^. In addition, few are the articles that still apply Machine Learning and Artificial Intelligence techniques to explain the behavior of smoking, something increasingly necessary given the increasingly complex environments in which tobacco control legislators operate^15-17^.

In this study, we investigated cigarette sales trends by brands after the two significant anti-tobacco policies (2005, 2010) in Spain, which include prohibitions on tobacco advertising, promotion, and sponsorship. We analyzed how different brands behaved concerning declining cigarette sales after comprehensive legislation. Although, to the best of our knowledge, this analysis has never been done from the perspective of brands, a recent study has analyzed the effect of anti-smoking laws from the perspective of convergence clubs^18^. To achieve the stated objective, we use the concept of ‘convergence club’. The premise of a ‘convergence club’ is that economic groups with similar initial characteristics, like the different brands of cigarettes that are sold in Spain, can realize a steady state of equilibrium through a balanced development path^19^. The idea is grounded in neoclassical growth models^20^ where the absolute or unconditional β-convergence hypothesis predicts countries as converging to a common steadystate equilibrium regardless of the initial conditions, whereas the conditional convergence hypothesis represents convergence to a common steady state, independent of the initial conditions and common structural properties^21^. Hence, this study aims at identifying whether there has been full convergence, divergence, or both, in cigarette sales in Spain from 2005 to 2021.

## METHODS

### Data collection

We obtained monthly sales data of tobacco products in Spain published by the Trade of Tobacco Commission of Spain (Commissioner for the Tobacco Market) from January 2005 to December 2021. In addition, data pre-processing was done to detect the brands that have been present on the market in the three sub-periods 2005–2021, 2005–2010 and 2011–2021. Thus, the 2005–2021 data panel has 8160 observations (40 brands with 204 months), the 2005–2010 data panel has 5328 observations (74 brands with 72 months), and the 2011–2021 data panel has 8844 observations (67 brands with 132 months). The data include all the brands that are marketed in Spain, so some will be cheap, others will not, some will be slim-fit cigarettes, others will not, etc. This heterogeneity should contribute to the richness of the study.

### Study design

For the development of this study, we used the methodology proposed by Phillips and Sul^22,23^ whose non-linear, time-varying factorial model is regarded the most advanced club detection model to date. This allows diverse temporal trajectories of the studied objects and their individual heterogeneity. This novel way of defining convergence clubs can determine, through the intrinsic characteristics of each object, its membership in a certain club, which, therefore, represents a great advantage in obtaining results. Other previous methods required the definition of a reference object *a priori*, and in a totally exogenous way, to classify the rest based on its position, which makes them less efficient and extrapolated.

### Data analysis

In this case, the formation of convergence clubs is given from the endogenous characteristics of each object and therefore the reliability, as well as its extrapolation, is greater^24^. To achieve the proposed objective, R Version 4.0 and the CovergenceClubs package have been used^24^. A more detailed elaboration of the methods can be found in the Supplementary file.

This technique has already been used previously to analyze the effectiveness of anti-smoking laws^18^, since it provides some advantages that can be summarized as: 1) it considers the average of the complete sample and measures its relative convergence, 2) it takes into account heterogeneities, which are based in a non-linear time-varying factorial model, 3) it considers heterogeneities, which depends on a non-linear time-varying factorial model, 4) it is robust to the unit root properties of the series, 5) the results are unbiased and consistent, and 6) eliminates the need for an ex-ante sample separation since it has a new algorithm based on data to determine the clusters of the subgroups of convergence.

## RESULTS

We present the results, according to the econometric strategy described, of the estimates to investigate the absolute and relative convergence in cigarette sales in Spain from 2005 to 2021. In the first step, we used log(t) test statistics to check if there is global convergence in the three selected periods according to the regulatory changes that have occurred in Spain. Second, once absolute convergence is rejected, we applied a clustering algorithm to test for the existence of subgroup convergence in the three selected periods.

The results of the log(t) test statistics are presented in Table 1, which shows the information necessary to check whether the null hypothesis of overall convergence can be rejected in the three selected periods. If we focus on the estimates made with the packs variable, Table 1 shows that the slope coefficient is equal to -0.747, -0.725, and -0.793, with heteroscedasticity and autocorrelation-consistent (HAC) standard error of 0.150, 0.026 and 0.139, for the three periods 2005–2021, 2005–2010 and 2011–2021, respectively. Furthermore, we obtain a t-statistic of -4.988, -28.041 and -5.702 (below the -1.65 critical value), respectively, for the three periods. Thus, the null hypothesis of overall convergence was rejected at the 5% significance level. These results suggest that cigarette sales in Spain by brands diverge or only converge among subgroups.

**Table 1:** Log(t) test statistics for cigarette sales by brands, 2005–2021

*Period*	*Number of brands*	*β*	*S.E.*	*t-statistic*	*p ^a^*
2005–2021	40	-0.747	0.150	-4.988	0.000***
2005–2010	74	-0.725	0.026	-28.041	0.000***
2011–2021	67	-0.793	0.139	-5.702	0.000***

a p-value associated with the convergence test (H0: convergence of sales).

*** The convergence hypothesis is rejected at 99% confidence.

** The convergence hypothesis is rejected at 95% confidence.

* The convergence hypothesis is rejected at 90% confidence. If no asterisks are marked, the hypothesis of convergence in cigarette sales cannot be rejected.

Next, we applied a clustering algorithm to test for the existence of subgroup convergence. The results (Tables 2–4) identify different clubs, and corresponding estimated β, where β=2α is the scaled coefficient of the speed of convergence of the club, and α is the estimated speed of convergence for any club^22^. As can be seen, the analysis of convergence subgroups has been carried out for the periods 2005–2021, 2005–2010 and 2011–2021. In all cases, the t-statistic > -1.65 critical value, implying within club convergence. This result holds for both the pre-2011 and post-2011 years. However, in the 2005–2010 period, 5 convergence clubs were observed, while in the 2011–2021 period the brands were grouped into 3 clubs. Analyzing the complete period (2005–2021) also 3 clubs are found. It seems that in the 2011–2021 period, cigarette sales have a more homogeneous behavior (3 clubs) than in the 2005–2010 period (5 clubs and 2 divergent brands), which supports previous literature that indicates that the Act 42/2010 provided a more restrictive specification of the ban than Act 28/2005.

**Table 2:** Convergence club process in cigarette sales by brand (in packs), 2005–2021

*Club*	*Number of brands*	*β*	*S.E.*	*t-statistic*	*p ^a^*	*Brands (N=40)*
1	17	0.793	0.262	3.026	0.9988	Marlboro, Camel, Chesterfield, Fortuna, Lucky Strike, Rothmans, Austin, Ducal, Karelia, Winston, L&M, Nobel, Ducados Rubio, Ducados Negro, Elixyr, R1, Benson & Hedges
2	16	0.264	0.081	3.251	0.9994	John Player SP., Bullbrand, Excite, Gauloises Rubio, BN, Vogue, Silk Cut, Winfield, Peter Stuyvesant, Burton, Pall Mall, News, Davidoff Rubio, Dunhill, Royal Crown, Gitanes
3	2	0.576	0.433	1.332	0.9086	Regal, Embassy
Divergent	5					Rex, Lambert & Butler, Mayfair, Superkings, Royals

a p-value associated with the convergence test (H0: convergence of sales).

*** The convergence hypothesis is rejected at 99% confidence.

** The convergence hypothesis is rejected at 95% confidence.

* The convergence hypothesis is rejected at 90% confidence. If no asterisks are marked, the hypothesis of convergence in cigarette sales cannot be rejected.

**Figure 1:**
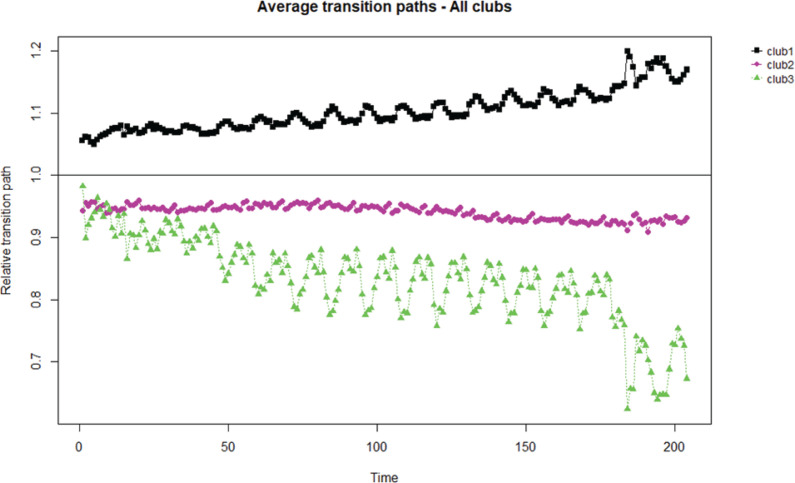
Convergence transition paths and speed in cigarette brands (N=40), 2005–2021

Table 2 and Figure 1 show the results of applying the clustering algorithm to test for the existence of subgroup convergence for the total period analyzed (2005–2021). As can be seen, the application of the algorithm generates 3 clubs and 5 marks as divergent. Of the 40 cigarette brands that have been marketed during this period, 17 and 16 belong to clubs 1 and 2, respectively, while club 3 only includes 2 brands. The transition path of club 1 is increasing, while in the case of club 2 a maintained transition path with a slight decreasing slope is observed. Club 3 only includes 2 brands with a high seasonal component due to their link with tourism.

**Figure 2:**
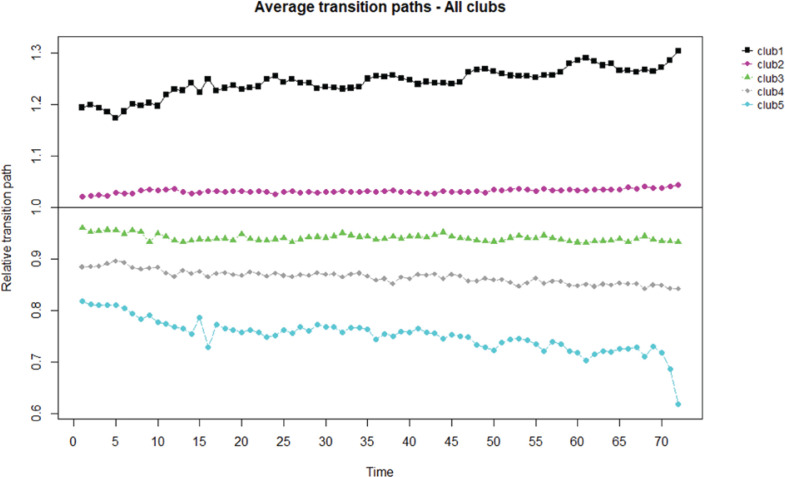
Convergence transition paths and speed in cigarette brands (N=74), 2005–2010

On the other hand, Table 3 and Figure 2 show the results of applying the clustering algorithm to test for the existence of subgroup convergence for the first subperiod analyzed (2005–2010). As can be seen, the application of the algorithm generates 5 clubs and 2 brands as divergent. Of the 73 cigarette brands that have been marketed during this period, 18, 28, 12 and 16 belong to clubs 1, 2, 3 and 4, respectively, while club 5 only includes 4 brands. It is in this subperiod that more heterogeneity is observed, something that seems reasonable according to previous studies on the effectiveness of anti-smoking laws in Spain, which indicate that Act 42/2010 was more effective than Act 28/2005. The transition path of club 1 is increasing, while in the case of clubs 2 and 3 a maintained transition path is observed. Clubs 4 and 5 show a decreasing trend in the transition paths.

**Table 3:** Convergence club process in cigarette sales by brand (in packs), 2005–2010

*Club*	*Number of brands*	*β*	*S.E.*	*t-statistic*	*p ^a^*	*Brands (N=74)*
1	12	0.034	0.19	0.18	0.5714	Marlboro, Winston, Fortuna, Lucky Strike, Pall Mall, L&M, Burton, Chesterfield, Camel, Ducados Negro, Ducados Rubio, Nobel
2	28	0.051	0.242	0.212	0.5839	John Player Sp., Elixyr, Gold Coast, R 1, Karelia, Ducal, Bn, Bullbrand, Philip ‘Morris, Excite, Austin, Gauloises Rubio, News, Lambert & Butler, Vogue, Habanos, Popular, West, Silk Cut, Peter Stuyvesant, Winfield, Golden American, Coronas Negro, Benson & Hedges, Next, Superkings, Royals, Lark
3	12	0.077	0.094	0.818	0.7932	Royal Crown, Celtas, Rex, Mayfair, Dunhill, Rothmans, Gitanes, Condal, Davidoff Rubio, Regal, Kool, Fine 120
4	16	0.108	0.151	0.712	0.7619	Reales, Brooklyn, Bisonte, Sovereign, Sombra, Boncalo, Berkeley, More, Embassy, Craven A, Prince, Partagas, Romeo Y Julieta, Piper, Belga, Diana
5	4	0.239	0.252	0.948	0.8284	Kensitas Club, Gold Leaf, Salem, Viceroy
Divergent	2					Coronas Rubio, Bastos

a p-value associated with the convergence test (H0: convergence of sales).

*** The convergence hypothesis is rejected at 99% confidence.

** The convergence hypothesis is rejected at 95% confidence.

* The convergence hypothesis is rejected at 90% confidence. If no asterisks are marked, the hypothesis of convergence in cigarette sales cannot be rejected.

**Table 4:** Convergence club process in cigarette sales by brand (in packs), 2011–2021

*Club*	*Number of brands*	*β*	*S.E.*	*t-statistic*	*p ^a^*	*Brands (N=67)*
1	28	0.244	0.029	8.479	0.9999	Marlboro, Camel, Chesterfield, Fortuna, Lucky Strike, Rothmans, Philip Morris K/S, Austin, Ducal, American Legend, Winston, L&M, Nobel, Ducados Rubio, Ducados Negro, Nobel Style, Elixyr, Marlboro Pocket, Fortuna Red Line, Lucky Strike Blando, Karelia, R 1, Bullbrand, Benson & Hedges, Nobel Blando, Excite, Denim, Manitou
2	30	-0.08	0.0584	-1.364	0.1568	Ducados Rubio Blando, John Player Sp., Benson & Hedges American, Gauloises Rubio, Bn, John Player Sp. Am.100’s, Ducados Rubio 100’s, Pueblo, Bravo, Vogue, John Player Sp. Black/Blue, Silk Cut, Winfield, Peter Stuyvesant, Burton, Pall Mall, News, Davidoff Rubio, Royal Crown, Dunhill, Desert Gold, Gitanes, Rex, Lambert & Butler, Natural American, Pepe, Gauloises Negro, Mayfair, Richmond, Superkings, Mohawk, John Player Sp. American, Natural American Spirit, Latino
3	9	-0.443	0.287	-1.544	0.1233	Richmond, Superkings, John Player Sp.American, Black Devil, Latino, Regal, Mecanicos, Embassy, Royals

a p-value associated with the convergence test (H0: convergence of sales).

*** The convergence hypothesis is rejected at 99% confidence.

** The convergence hypothesis is rejected at 95% confidence.

* The convergence hypothesis is rejected at 90% confidence. If no asterisks are marked, the hypothesis of convergence in cigarette sales cannot be rejected.

**Figure 3:**
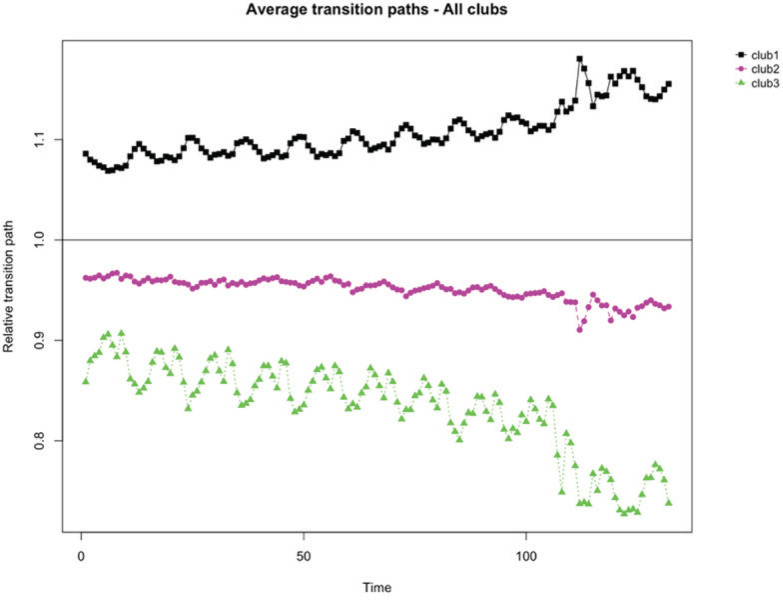
Convergence transition paths and speed in cigarette brands (N=67), 2011–2021

Finally, Table 4 and Figure 3 show the results of applying the clustering algorithm to test for the existence of subgroup convergence for the second analyzed subperiod (2011–2021). In this case, the application of the algorithm generates 3 clubs. Of the 67 cigarette brands that have been marketed during this period, 28 and 30 belong to clubs 1 and 2, respectively, while club 3 includes 9 brands. The transition path of club 1 is increasing, while in the case of clubs 2 and 3 a decreasing transition path is observed.

Thus, cigarette sales by brands are not one homogeneous convergence club; they constitute heterogenous clubs of different sizes, on different transition paths and speeds. This is a non-trivial insight because it suggests that whereas the null hypothesis of overall convergence was rejected, within this same group there is evidence of both convergence and non-convergence.

## DISCUSSION

In recent years, there has been a growing interest among researchers, practitioners, and policymakers in finding economic policy tools to restrict tobacco consumption. Control is crucial not only due to the large impact that tobacco consumption has on health, but also to its effect on the budgets of countries via tax collection and health costs. Tobacco advertising, promotion and sponsorship can influence tobacco consumption. In this context, the empirical literature devoted to the analysis of the effectiveness of economic policy tools is as heterogeneous as the effects of the ban on advertising, promotion, and sponsorship on tobacco consumption. In addition, few studies analyze the effect of two laws that restrict tobacco advertising, promotion, and sponsorship from a cigarette sales perspective, by brand. In this article, the effect of the 2005 and 2010 restrictions on the evolution of cigarette sales by brand has been explored in a novel way that allows an analysis of the convergence in sales of all brands marketed in Spain.

By applying techniques that test for convergence clubs, we reveal that the laws of 2005 and 2010 have not managed to homogenize the market by promoting absolute convergence. Although the existence of absolute convergence is rejected for all the periods studied, we find different effects of the 2005 and 2010 laws. Our results suggest that in Spain, from 2005 to 2010 brand sales behaved more heterogeneously than from 2010, where, although there is no absolute convergence, only 3 clubs are found. This finding supports what is established in the previous literature that indicates that Act 42/2010 provided a more restrictive specification of the ban than Act 28/2005. However, it seems that the convergence or neutrality sought by the government has not been achieved, something consistent with a recent study that indicates that ‘tobacco companies have taken advantage of the loopholes to continue promoting their products’^25^.

Although the ban on tobacco advertising, promotion and sponsorship in Spain has not generated absolute homogenization or neutrality, there is heterogeneous behavior in the different sub-periods. On the one hand, the Act 28/2005 did not prevent tobacco companies from differentiating their brands and, therefore, the behavior of sales was very heterogeneous. On the other hand, the 2011 law, which includes a more restrictive specification of the ban than Act 28/2005, although it has not achieved absolute convergence, has produced more homogenization. This seems to show the effectiveness of restrictions on tobacco advertising, promotion, and sponsorship. According to these results, it seems that two hypotheses are fulfilled: the point of view of Spanish regulation and the economic theory associated with the regulation of goods that cause harm to health. On the one hand, the 2011 law has been more effective than Act 28/2005. On the other hand, the results suggest that the more restrictive the regulation on tobacco advertising, promotion and sponsorship, the more homogenization or neutrality is achieved.

To summarize, the practical policy implications that the results of this study have for policymakers, can be summarized in three. The first implication is that governments have in the laws an instrument to control legal cigarette sales. The more restrictive the limitations on tobacco advertising, promotion, and sponsorship, the more limited the ability of tobacco manufacturers to differentiate themselves in the market. Secondly, policymakers should consider that restrictions on tobacco advertising, promotion, and sponsorship, while limiting the ability of manufacturers to differentiate themselves, do not bring about full convergence in which all brands are ‘one size fits all’ for consumers. Finally, it seems that the laws that include limitations on tobacco advertising, promotion and sponsorship generate a kind of ‘freezing effect’ that provides advantages to the best-positioned brands just now in which the laws come into force.

### Implications

A natural extension of the present work for future research would be to test the convergence of the substitute products for cigarettes, which would give an answer as to whether the greatest convergence observed from 2010 is related to the behavior of other substitute products, which include legal tobacco products, such as fine-cut tobacco or pipe tobacco, and even illegal products. Furthermore, another extension may be to analyze if the non-convergence observed in this study is different, depending on the interest group (such as young people, men, women or the unemployed, for example). Another future line may be to include the effect of the cross-border trade and smuggling. Finally, a spatial analysis that allows studying the convergence by regions can also add value to the existing literature.

### Limitations

The results of this study are not without limitations. First, although the cigarette market accounts for approximately 90% of the total tobacco market in Spain during the period analyzed, the effect that substitute products may have had on this convergence has not been considered. A recent article indicates that some tobacco manufacturers in Spain are using substitute products such as heated tobacco products as an alternative to cigarettes^26^. Secondly, it has not been considered that there are provincial anomalies in Spain that must be considered when analyzing official data^27^. In addition, macro data have been used that do not allow the analysis of individual behaviors. Finally, spatial analysis techniques have not been used, something important in Spain, given the high and low consumption clusters that exist at the provincial level^28^.

## CONCLUSIONS

The action of the Spanish government to control the sale and promotion of cigarettes has produced the desired effect. Although the partial limitation law caused a standardization effect on cigarette sales, sales began to behave in the same way after the law that totally limited advertising, promotion, and sponsorship. Therefore, the law of total limitation has been very effective in preventing brands from differentiating themselves by carrying out marketing actions.

## Data Availability

The data supporting this research are available from the authors on reasonable request.
